# Jasmonic Acid and Salicylic Acid Crosstalk Mediates Asymmetric Interactions Between *Aphis gossypii* and *Lema decempunctata* in *Lycium barbarum*

**DOI:** 10.3390/insects16090876

**Published:** 2025-08-23

**Authors:** Zhongxu Liu, Beibei Zhu, Changrong Deng, Guozhen Duan, Jianling Li, Guanghui Fan

**Affiliations:** Qinghai Plateau Tree Genetics and Breeding Laboratory, Laboratory for Research and Utilization of Qinghai Tibet Plateau Germplasm Resources, Academy of Agriculture and Forestry Sciences, College of Agriculture and Animal Husbandry, Qinghai University, Xining 810016, China; liuzhongxu3019@163.com (Z.L.); 15249360623@163.com (B.Z.); dengchang_rong@126.com (C.D.); 18848110959@163.com (G.D.); qhfgh@163.com (G.F.)

**Keywords:** aphid, beetle, goji berry, plant-mediated interaction, hormone

## Abstract

The aphid, *Aphis gossypii* Glover, and the leaf beetle, *Lema decempunctata* Gebler, are two catastrophic pests affecting the production of the organic goji berry, *Lycium barbarum* L. Our previous studies have demonstrated that the defense responses of goji berry induced by aphid infestations can facilitate the growth and development of beetles. However, the reciprocal effects of these two insect infestations on aphids remained unclear. In this study, the impacts of these two pest infestations on the development, survival, and reproduction of aphids were examined. Additionally, the levels of plant defense-related hormones, salicylic acid (SA) and jasmonic acid (JA), were measured. Subsequently, host plants were treated with two hormone analogues, 2, 1, 3-benzothiadiazole (BTH) and methyl jasmonate (Me-JA), to identify their effects on aphid development, survival, and reproduction. The results showed that the total developmental duration was accelerated by 33.60%, and the total reproduction of aphids was increased by 82.98% compared to the control after aphid infestation, without influencing survival. In contrast, the beetle infestation did not significantly influence any aspect of the aphid population. The content of SA in plants after the aphid infestation and JA after the beetle infestation increased 19.42 times and 400.50 times, respectively, compared with the control. The total developmental duration of aphids treated with BTH was reduced by 13.44%, while their reproduction increased by 60.52% compared with the control. The total developmental duration of aphids treated with Me-JA was prolonged by 23.51% compared to the control, while survival rates and reproduction were unchanged. Our research elucidates the intricate interspecific relationship between *A. gossypii* and *L. decempunctata*, providing valuable insights into the complex interspecific relationship between the two pests and informing effective strategies for their scientific prevention and control.

## Simple Summary

*Aphis gossypii* Glover and *Lema decempunctata* Gebler are two catastrophic pests affecting the production of organic *Lycium barbarum* L. Clarifying the intricate interspecific relationship between the two pests is of significance for their scientific control. In this study, the impacts of these two pest infestations on the development, survival, and reproduction of aphids were examined. The results showed that aphid infestation significantly promoted its own development and reproduction without influencing survival. In contrast, beetle infestation did not significantly affect any aspect of the aphids. The salicylic acid (SA) content in plants significantly increased following aphid infestation, whereas the jasmonic acid (JA) content exhibited a marked increase after beetle infestation. Host plants treated with 2, 1, 3-benzothiadiazole (BTH) facilitated the development and reproduction of aphids, while treatment with methyl jasmonate (Me-JA) inhibited their development.

## 1. Introduction

Interspecies interactions play a crucial role in shaping the behaviors, distributions, populations, and community structures of herbivorous insects [1]. These interactions encompass competition, predation, parasitism, mutualism, commensalism, and neutral interactions [2]. Factors that mediate interspecies interactions include host plants, natural enemies, climate, and other factors, among which host plants exert the most significant influence [1]. After being infested by insects, plants typically activate signaling pathways, such as the salicylic acid (SA) pathway or the jasmonic acid (JA) pathway, to synthesize toxic compounds or modulate nutrient availability as defensive mechanisms against insects [3]. Therefore, insects that share the same host plant and compete for limited food resources will indirectly affect the growth, development, selection, and reproduction of other herbivores by inducing plant defenses. Most of these effects are detrimental, with only a few being beneficial [2].

Salicylic acid (SA) and iasmonic acid (JA) are two key defense hormones that play critical roles in both basal and induced resistance against a wide range of harmful organisms [4]. Since the stylets of phloem-sucking insects bypass epidermal cells and mesophyll cells during feeding, the damage inflicted on plant tissues is relatively localized [5]. The defense response elicited by this feeding behavior resembles that triggered by biotrophic pathogens, typically activating the plant’s salicylic acid (SA) signaling pathway [6,7,8]. However, chewing mouthpart insects inflict mechanical damage on plants through their biting actions, which can lead to significant tissue destruction [9,10]. The plant defense mechanisms elicited by this feeding behavior are analogous to those triggered by necrotrophic pathogens, primarily involving jasmonic acid (JA) signaling pathways [11,12].

Many studies have indicated that the SA signaling pathway and JA signaling pathway exhibit antagonistic interactions [13,14,15,16]. When phloem-sucking insects infest plants, the SA signaling pathway is activated, while the JA signaling pathway is suppressed. Conversely, an opposite response occurs during attacks by chewing insects [17,18]. In the SA signaling pathway, NPR1 represses the interactions of MYC2, MYC3, and MYC4 with the mediator complex MED25 in the JA signaling pathway [19], thereby suppressing the expression of JA-responsive genes such as *LOX2* and *VSP2* [20]. In contrast, the COI1 in the JA signaling pathway can inhibit the expression of pathogenesis-related (*PR*) genes in the SA pathway by suppressing WRKY70 [21]. Unlike JA-mediated defense responses, SA-mediated defense responses usually do not exert significant inhibitory effects on the growth and development of insects. Therefore, the induction of SA by phloem-sucking insects in the host plant, which inhibits the synthesis and transduction of JA, is considered a strategy employed by these insects to evade effective plant defenses [17].

Methyl jasmonate (Me-JA) is the primary volatile derivative of JA and functions as a key inducer of plant defense responses. Elevated levels of Me-JA activate JA-responsive genes, such as vegetative storage protein (*VSP*) and plant defensin 1.2 (*PDF1.2*) [22]. The defense mechanisms triggered by chewing insects closely resemble those elicited by exogenous application of Me-JA. However, Me-JA does not exert direct insecticidal effects; rather, it acts as a plant immune elicitor, inducing changes in the major defense-related substances in plant [23]. 2, 1, 3-Benzothiadiazole (BTH), a synthetic analog of SA, can induce defense responses typically associated with biotrophic pathogens and is commonly referred to as a plant activator. Notably, BTH lacks direct antimicrobial activity in vitro [24,25]. Both BTH and SA utilize similar cis-acting regulatory elements to activate the PR-1a promoter, thereby enhancing the expression of SA biosynthesis-related genes such as phenylalanine ammonia-lyase (*PAL*), initiating signal transduction pathways involved in SA accumulation or downstream systemic acquired resistance (SAR) [26].

The phloem-sucking aphid, *Aphis gossypii* Glover, and the chewing beetle, *Lema Decempunctata* Gebler, are two major pests affecting goji berr, *Lycium barbarum* L., frequently coexisting and feeding on the plant’s foliage. Our previous studies have demonstrated that *A. gossypii* infestation facilitates the growth and development of *L. decempunctata* while also exhibiting an attractive effect on beetle feeding and oviposition [27]. However, beetle infestation has a detrimental impact on its own development [27]. Therefore, the facilitative effects of the aphid infestation on these beetles may contribute significantly to the extensive damage caused by the beetles to goji berr. Nevertheless, the effects of aphid and/or beetle infestation on *A. gossypii* development, survival, and reproduction remain unclear. Moreover, although *A. gossypii* is frequently observed aggregating on the tender shoots of goji berr, the functional significance of this behavior for its growth and development has not been determined. Additionally, while aphid infestation has been demonstrated to promote beetle development, it remains unclear whether beetles reciprocally facilitate aphid development. Understanding these issues is of great significance for comprehensively elucidating the complex interspecies interactions between *A. gossypii* and *L. decempunctata*, revealing the mechanisms underlying the severe damage and outbreak-prone nature of aphids and providing guidance for scientific control.

## 2. Materials and Methods

### 2.1. Experimental Plants and Herbivores

Breeding of goji berry seedlings: In July 2023, mature fruits of goji berry (*L. barbarum*, variety ‘Ningqi 1’) were harvested from Nuomuhong Farm, Haixi Prefecture, Xining City, China (96°15′ E, 36°20′ N). These fruits were dried in the sun and then soaked in water for 5 h. Through repeated manual rubbing, shriveled seeds and debris that floated to the surface were discarded, while plump seeds that sank to the bottom were retained. These seeds were then air-dried under indoor conditions. Seeds were sown into 28-cell seedling trays containing nursery substrate (Xinyinong Agricultural Science and Technology Co., Ltd., Xin County, Liaocheng, Shandong, China) and cultivated in a light incubator (25 ± 2 °C, 70 ± 5% RH, and a photoperiod of L16 h: D8, HGP-860, Qingdao Haier Bio-Medical Technology Co., Ltd., Qingdao, Shandong, China) at Qinghai University (96°25′ E, 36°26′ N). Seedlings with a height of approximately 5 cm were transplanted into plastic cups (volume: 6.5 × 6.5 × 9 cm) and cultivated under the environmental conditions mentioned above. Seedlings were considered suitable for subsequent use when they reached a height of 15 cm, at an approximate age of 30 days.

Breeding of *A. gossypii*: Aphids was collected from the organic goji berry base at Nomuhong Farm (96°15′ E, 36°20′ N) and reared using goji berry seedlings under the conditions mentioned above.

Breeding of *L. decempunctata*: Leaves with beetle eggs were collected from the organic goji berry base at Nomuhong Farm. These leaves were then placed into petri dishes with moist filter paper and incubated under the conditions mentioned above. When the eggs hatched into larvae, they were transferred to colorless transparent plastic containers (750 mL) containing 3 cm of moist sand at the bottom. Young goji berry branches were placed and replaced daily. The second instar larvae were selected for the subsequent research.

### 2.2. Experimental Methods

#### 2.2.1. Investigation of the Growth, Development, Survival, and Reproduction of *A. Gossypii* Under Elicitor Treatments

**Figure 1:**
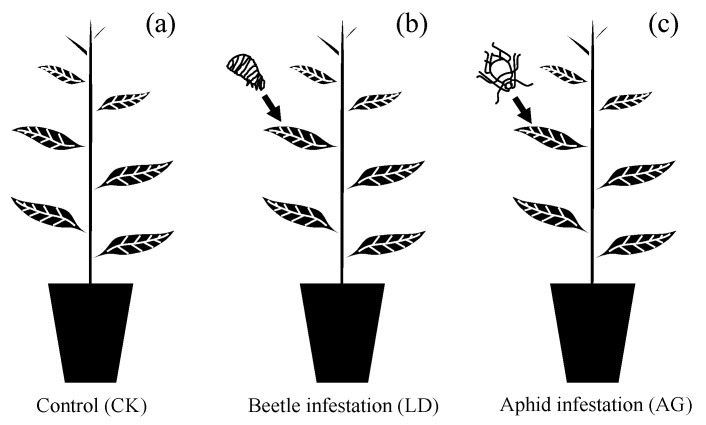
Experimental design for the three treatments. (**a**): CK (control) group, not inoculated with herbivores. (**b**): LD group, inoculated with one 2nd *L. decempunctata* larvae. (**c**): AG group, inoculated with 30 3rd instar *A. gossypii* nymphs.

Three treatments were conducted: Control (CK), *L. decempunctata* infestation (LD), and *A. gossypii* infestation (AG). In the CK treatment, seedlings were not inoculated with any herbivores (Figure 1). In the LD treatment, one 2nd instar *L. decempunctata* larva was inoculated onto the middle leaf of the seedling and removed after 48 h. In the AG treatment, thirty 3rd instar *A. gossypii* nymphs were inoculated onto the middle leaf of the seedling and subsequently removed after 48 h.

One 1st instar neonatal aphid was meticulously inoculated onto the tip of the seedling in all treatments. The instar and the survival of the aphids were recorded for ten consecutive days until they matured into adults. Then, nymphs propagated by the adult aphids were meticulously removed daily, and their numbers were recorded for seven consecutive days. Eighteen seedlings were used in each treatment. All seedlings were cultivated under the conditions mentioned above.

#### 2.2.2. Quantitative Investigation of JA and SA in Goji Berry Under Elicitor Treatments

The CK, LD, and AG treatments were conducted as described in Section 2.2.1. After 48 h of infestation, these pests were removed from the seedlings, and 500 mg of foliage was collected per treatment to determine the contents of JA and SA following the methodology outlined by Yang et al. [28]. Each treatment was replicated three times, with each replication consisting of five seedlings. All seedlings were cultivated under the conditions mentioned above. The phytohormone extraction and quantification protocols are as follows:

JA and SA content were measured by MetWare (http://www.metware.cn/, accessed on 19 September 2024) (Wuhan, China) based on the AB Sciex QTRAP6500 LC-MS/MS platform. The leaves were homogenized and ground into a fine powder, and 50 mg was accurately weighed, then dried using a freeze-vacuum dryer. Finally, the powder was extracted with 0.5 mL of a combination solution of n-hexane, acetone, and ethanol (1:1:1, *v*/*v*/*v*), which included 0.1% (*v*/*v*) butylated hydroxytoluene (BHT). The extract was vortexed at room temperature for 20 min. After centrifuging at 12,000 rpm for 5 min at 4 °C, the supernatants were collected, and the extraction process was conducted twice. Afterward, it was dried via evaporation before being reconstituted in a 1:1 *v*/*v* mixture of methanol (MeOH) and methyl tert-butyl ether (MTBE) mixtures. For further liquid chromatography–tandem mass spectrometry (LC-MS/MS) analysis, the solution was filtered through a 0.22 μm filter. A UPLC-APCI-MS/MS system (UHPLC, ExionLCTMAD; MS, Applied Biosystems 6500 Triple Quadrupole, Waltham, MA, USA) was then used to analyze the sample extracts.

#### 2.2.3. Investigation of the Effects of Me-JA and BTH Application on the Growth, Development, Survival, and Reproduction of *A. gossypii*

Three treatments were performed: Control (CK), methyl jasmonate (Me-JA), and 2, 1, 3-Benzothiadiazole (BTH) treatments. CK treatment: 2 mL of anhydrous ethanol (Shanghai Macklin Biochemical Technology Co., Ltd., 99.5%, Shanghai, China) was diluted with distilled water to a final volume of 200 mL, and 10 mL of the solution was sprayed on the seedlings; Me-JA treatment: 88.88 μL of methyl jasmonate (Me-JA) stock solution (Shanghai Macklin Biochemical Technology Co., Ltd., 98%, Shanghai, China) was initially diluted with 2 mL of anhydrous ethanol and distilled water was added to make the final volume 200 mL to prepare a 2 mmol/L Me-JA solution [23], then 10 mL was sprayed on the seedlings; BTH treatment: 0.02 g of 2, 1, 3-benzothiadiazole (BTH) (Shanghai Macklin Biochemical Technology Co., Ltd., 99%, Shanghai, China) was dissolved in 2 mL of anhydrous ethanol, then distilled water was added to make the final volume 200 mL to prepare a 0.7 mmol/L BTH solution [29,30], then 10 mL was sprayed on the seedlings. After 24 h, the seedlings from each treatment were inoculated with one 1st instar neonatal aphid. The survival and developmental stages of the aphids were recorded daily until they developed into adults. Subsequently, the number of nymphs produced by adult aphids was recorded, and they meticulously removed daily for seven consecutive days. Each treatment was replicated 20 times, with each replication consisting of one seedling. All seedlings were cultivated under the conditions mentioned above.

#### 2.2.4. Statistical Analysis

The data were analyzed using the statistical software SPSS v27 (IBM, Armonk, NY, USA). Significant differences in the development duration, the reproduction, and the content of JA and SA were assessed using a one-way ANOVA followed by the least significant difference (LSD) tests. The survival of *A. gossypii* under elicitor treatments was evaluated using the Chi-square test. The number of nymphs produced by adult aphids at various times in elicitor treatments was analyzed using a two-factor repeated measures ANOVA followed by a LSD test.

## 3. Results

### 3.1. Effects of L. decempunctata or A. gossypii Infestation on the Growth, Development, Survival, and Reproduction of Aphids

**Figure 2:**
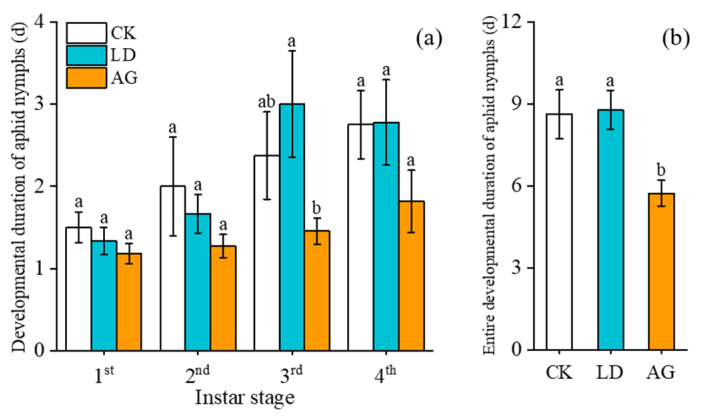
Effects of elicitor treatments on (**a**) the development duration of different instar aphid nymphs and (**b**) the total developmental duration of aphid nymphs (Mean ± SE). Different letters above the bars indicate significant differences (*p* < 0.05); identical letters above the bars indicate no significant differences *(p* > 0.05).

There were no significant differences in the developmental duration of 1st, 2nd, and 4th instar aphid nymphs among the three treatments (Figure 2a). However, the developmental duration of 3rd instar nymphs in AG was reduced by 51.67% compared to that in LD (*F***_2,25_** = 3.115, *p* = 0.021). No significant differences were observed between CK and LD (*F***_2,25_** = 3.115, *p* = 0.365) (Figure 2a).

The total developmental duration of *A. gossypii* nymphs in AG was 5.73 d, representing a reduction of 33.60% (*F*_2,25_ = 7.160, *p* = 0.005) and 34.74% (*F*_2,25_ = 7.160, *p* = 0.003) compared to CK and LD, respectively. No significant differences were found between CK and LD (*F*_2,25_ = 7.160, *p* = 0.879) (Figure 2b).

**Figure 3:**
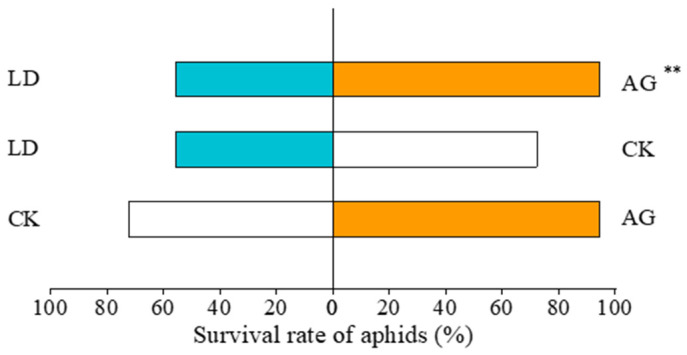
Effects of elicitor treatments on the survival of aphids. ** indicates significant differences (0.001 < *p* < 0.01).

The survival rate of *A. gossypii* in AG was 94.44%, with an increase of 69.98% compared to that in LD (*χ*^2^ = 7.259, *p* = 0.007). No significant differences were observed between AG and CK (*χ*^2^ = 3.200, *p* = 0.074). The survival rate of *A. gossypii* in LD was 55.56%, with no significant differences compared to that in CK (*χ*^2^ = 1.084, *p* = 0.298) (Figure 3).

**Figure 4:**
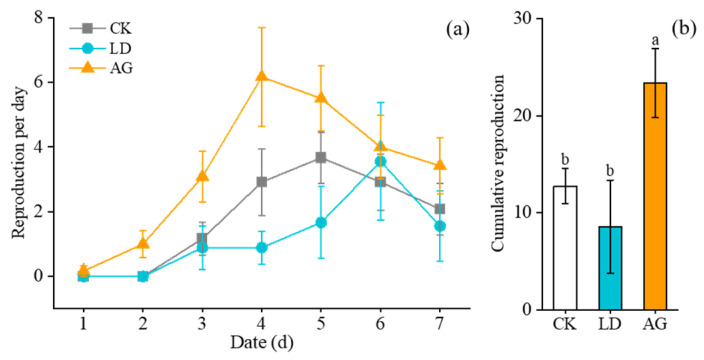
(**a**) Reproduction of aphids per day under elicitor treatments; (**b**) Cumulative reproduction of aphids under elicitor treatments (Mean ± SE). Different letters above the bars indicate significant differences (*p* < 0.05); identical letters above the bars indicate no significant differences (*p* > 0.05).

The reproduction of aphids under elicitor treatments exhibited an initial increase followed by a subsequent decrease (Figure 4a). Significant differences were observed in aphid reproduction over time (*F*_6,25_ = 11.272, *p* < 0.001) and among treatments (*F*_2,30_ = 5.013, *p* = 0.013). However, the interaction between time and treatment was not significant (Time × Treatments: *F*_12,52_ = 1.492, *p* = 0.179), indicating that the impact of treatments on aphid reproduction remained consistent over time.

The total reproduction of aphids under the AG treatment was 23.33 offspring per seedling, which was increased by 172.55% (*F*_2,30_ = 5.013, *p* = 0.006) and 82.98% (*F*_2,30_ = 5.013, *p* = 0.028) compared to the LD and CK treatments, respectively. The total reproduction in aphids under the LD treatment decreased by 32.86% in comparison to the CK treatment; however, this difference was not statistically significant (*F*_2,30_ = 5.013, *p* = 0.403) (Figure 4b).

### 3.2. Effects of L. decempunctata or A. gossypii Infestation on the Content of JA and SA in Goji Berry

**Figure 5:**
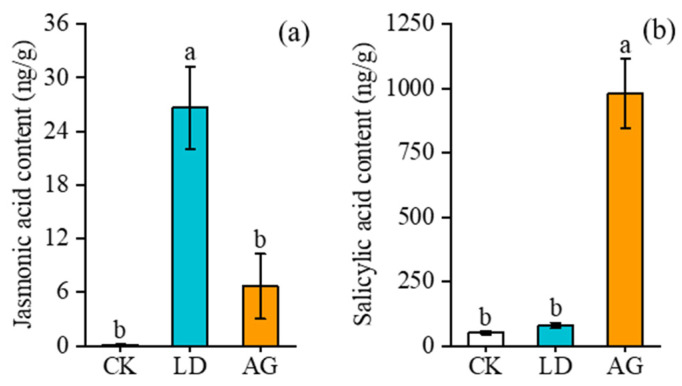
Effects of *L. decempunctata* or *A. gossypii* infestation on the content of (**a**) JA and (**b**) SA in goji berry (mean ± SE). Different letters above the bars indicate significant differences (*p* < 0.05); conversely, identical letters above the bars indicate no significant differences (*p* > 0.05).

The JA content in the AG treatment was 6.67 ng/g, which was significantly lower than that in the LD treatment (*F*_2,6_ = 16.536, *p* = 0.006), while no significant difference was observed compared to CK (*F*_2,6_ = 16.536, *p* = 0.219). The JA content in the LD treatment was 26.63 ng/g, which increased 400.50 times compared to CK (*F*_2,6_ = 16.536, *p* = 0.001) (Figure 5a).

In contrast, the SA content under the AG treatment reached 979.86 ng/g, which increased 19.42 times and 12.39 times compared to CK (*F*_2,6_ = 45.986, *p* < 0.001) and LD (*F*_2,6_ = 45.986, *p* < 0.001), respectively. No significant differences were found between CK and LD (*F*_2,6_ = 45.986, *p* = 0.804) (Figure 5b).

### 3.3. Effects of Me-JA and BTH on the Growth, Development, Survival, and Reproduction of A. gossypii

**Figure 6:**
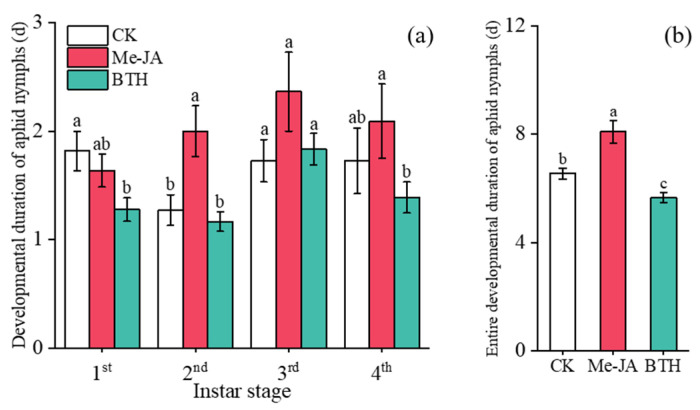
Effects of elicitor treatments on (**a**) the developmental duration of aphid nymphs at different instar stages and (**b**) their entire developmental duration (mean ± SE). Different letters above the bars indicate significant differences (*p* < 0.05); identical letters above the bars indicate no significant differences (*p* > 0.05).

The developmental duration of different instar aphid nymphs is shown in Figure 6a. Under the BTH treatment, the developmental duration of 1st instar nymphs was reduced by 29.67% compared to CK (*F*_2,37_ = 4.122, *p* = 0.009). However, no significant differences were observed when comparing it to Me-JA (*F*_2,37_ = 4.122, *p* = 0.077). The developmental duration of 2nd instar nymphs under the Me-JA treatment was prolonged by 57.48% and 70.94% when compared to CK (*F*_2,37_ = 8.821, *p* = 0.003) and BTH (*F*_2,37_ = 8.821, *p* < 0.001), respectively. There was no significant difference between BTH and CK (*F*_2,37_ = 8.821, *p* = 0.609). There were no significant differences detected in the developmental duration of 3rd instar nymphs across these treatments. The developmental duration of 4th instar nymphs under the BTH treatment was shortened by 33.49% in comparison to Me-JA (*F*_2,37_ = 2.146, *p* = 0.047). Furthermore, no significant difference was observed when compared with CK (*F*_2,37_ = 2.146, *p* = 0.327) (Figure 6a).

The entire developmental duration of aphid nymphs under the BTH treatment was 5.67 d, indicating a reduction of 13.44% (*F*_2,37_ = 20.837, *p* = 0.025) and 29.91% (*F*_2,37_ = 20.837, *p* < 0.001) when compared to CK and Me-JA, respectively (Figure 6b). In contrast, the entire developmental duration of aphid nymphs under the Me-JA treatment was 8.09 d, representing an extension of 23.51% in comparison to CK (*F*_2,37_ = 20.837, *p* = 0.001) (Figure 6b).

**Figure 7:**
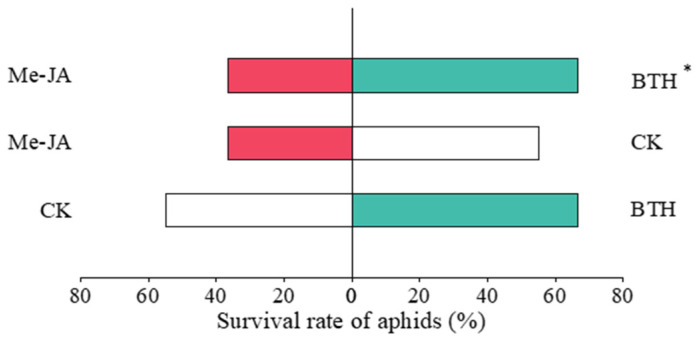
Effects of elicitor treatments on the survival of aphids. * above the bars indicates statistically significant differences (*p* < 0.05).

The survival rate of *A. gossypii* under the BTH treatment was 66.67%, representing an increase of 81.81% compared to Me-JA (*χ*^2^ = 5.406, *p* = 0.02). However, there were no significant differences between the BTH and CK treatments (*χ*^2^ = 0.693, *p* = 0.405). Additionally, the survival rate of *A. gossypii* under the Me-JA treatment did not differ significantly from CK (*χ*^2^ = 1.637, *p* = 0.201) (Figure 7).

**Figure 8:**
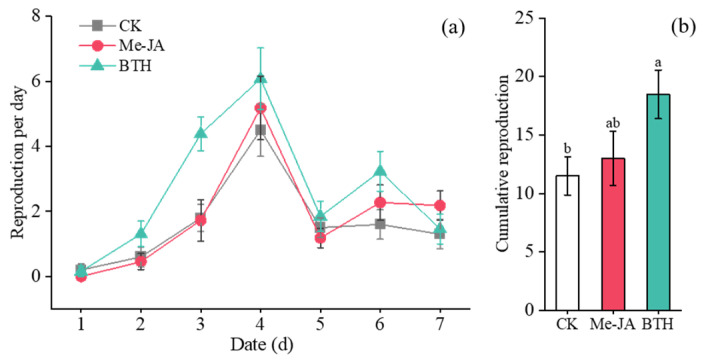
(**a**) Reproduction of aphids per day under elicitor treatments; (**b**) Cumulative reproduction of aphids under elicitor treatments (mean ± SE). Different letters above the bars indicate significant differences (*p* < 0.05); identical letters above the bars indicate no significant differences (*p* > 0.05).

The reproduction of aphids under the various treatments demonstrated an initial increase, followed by a subsequent decline (Figure 8a). There were significant differences in aphid reproduction over time (time: *F*_6,26_ = 36.276, *p* < 0.001) and among treatments (Treatments: *F*_2,31_ = 3.300, *p* = 0.05). However, no interactions were found between time and treatments (Time × Treatments: *F*_12,54_ = 1.716, *p* = 0.120), indicating that the effects of the treatments on aphid reproduction did not change over time.

The entire reproduction of aphids under the BTH treatment was 18.46 offspring per seedling, representing an increase of 60.52% (*F*_2,31_ = 3.300, *p* = 0.023) and 42.00% (*F*_2,31_ = 3.300, *p* = 0.064) compared to the CK and Me-JA treatments, respectively. However, there was no significant difference between the Me-JA and CK treatments (*F*_2,31_ = 3.300, *p* = 0.624) (Figure 8b).

## 4. Discussion

The damage caused by phloem-sucking insect to plants is always limited, and this feeding behavior often triggers the SA signaling defense [31]. Due to the antagonistic interactions between the SA and JA signaling pathways, the activation of plant defenses mediated by SA in response to phloem-sucking insects typically leads to the suppression of the JA signaling pathway [32]. Our research findings demonstrated that following aphid infestation, the content of SA in plants significantly increased, while the levels of JA remain unchanged. Previous studies have confirmed that activation of the SA signaling pathway typically suppresses catalase activity in plants, resulting in an increase in endogenous H_2_O_2_ [33]. And, it binds to SABP3, which possesses antioxidant activity, inhibiting its activity and resulting in the accumulation of reactive oxygen species (ROS) [34]. This can trigger the upregulation of plant defense-related genes and can also directly inhibit the growth of pathogens as a toxic compound [35], thereby enhancing plants’ resistance to bacterial and viral pathogens [36]. The activation of the JA signaling pathway leads to the synthesis of a large number of JA signaling molecules in the plant, which in turn form JA-Ile or other amino acid derivatives. Subsequently, JAZ proteins are degraded, initiating the transcription of JA-responsive genes [37]. This causes plants to synthesize defense-related proteins, such as protease inhibitors (PIs) and polyphenol oxidases (PPOs), along with toxic secondary metabolites, including alkaloids and tannins, thereby inhibiting the growth and development of insect herbivores [38]. Therefore, the behavior of phloem-feeding insects—activating the SA signaling pathway while concurrently inhibiting the JA signaling pathway—is considered an evasive strategy against effective plant defense [39]. For example, infestation of *Arabidopsis thaliana* (L.) Heynh mutants by *Bemisia tabaci* (Gennadius) showed that mutants with activated SA defenses (*cim10*) or impaired JA defenses (*coi1*) accelerated the nymphal development of *B*. *tabaci*; conversely, mutants with activated JA defenses (*cev1*) or impaired SA defenses (*npr1*) decelerated *B. tabaci* nymphal development [40]. Our research demonstrated that when *A. gossypii* feed on plants that they had previously infested or on those treated with exogenous SA analogues, their development was accelerated, and their reproduction capacity was enhanced. During the development process of each instar of the aphids, we found that the developmental duration of aphids under elicitor treatments only showed significant differences at the 3rd instar. This might be due to the fact that we investigated the developmental duration of aphids once a day, which might have missed the molting time of the aphids.

The feeding behavior of insects with chewing mouthparts causes significant damage to plants and activates the JA signaling pathway [31]. The results of our study showed a notable increase in JA content in *L. decempunctata*-infested plants, while the SA content did not exhibit any significant changes. Upon activation of the JA signaling pathway, plants increase the production of various defense proteins and secondary metabolites, such as alkaloids and phenolics, which play crucial roles in deterring or inhibiting herbivore attacks [41]. Although our results indicate that *L. decempunctata* feeding has no significant effect on the development, reproduction, or survival of *A. gossypii*, the application of exogenous Me-JA results in a prolonged developmental duration of the aphid. This phenomenon might be caused by the different feeding positions of the two pests. Although we inoculated the leaf beetles and aphids at the middle leaf position of the seedlings, the leaf beetles predominantly fed at the inoculation site, whereas the aphids tended to migrate to the tender shoots for feeding. Previous research has demonstrated that the JA-mediated defense response triggered by chewing herbivores is often localized and does not elicit systemic defense reactions in other parts of the plant [42,43]. As reported by Zhang et al. [44] and Floková et al. [45], JA and its precursor compounds primarily accumulate in the wounded regions rather than in the undamaged areas. Therefore, we hypothesized that the *L. decempunctata* feeding-induced JA signaling pathway may also serve as a localized defense, which did not affect the growth and development of pests in other regions. However, after treating goji berry seedlings with exogenous Me-JA, we observed that the development of *A. gossypii* was decreased. This phenomenon may be attributed to the exogenous application of JA throughout the entire plant, triggering a systemic defense response within the plant. For example, treatment of *Sorghum bicolor* L. with exogenous Me-JA activated the plant defense response, which prevented the invasion of *Schizaphis graminum* (Rondani) [46]. However, local application of JA to the basal leaves of *Chrysanthemum morifolium* Ramat. marginally affected the metabolomic profiles of systemic non-treated apical leaves and resulted in no significant resistance to *Frankliniella occidentalis* (Pergande) [47].

However, after exogenous application of Me-JA, there was no effect on the reproduction of aphids, potentially because most substances are synthesized in the cytoplasm, the ER (endoplasmic reticulum), or organelles. Hydrophilic secondary metabolites are usually stored in the vacuole after their formation in cytoplasm, whereas lipophilic substances are sequestered in resin ducts, laticifers, glandular hairs, trichomes, thylakoid membranes, or on the cuticle [48]. However, aphids pierce through the cuticle and bypass the epidermal cells and mesophyll cells during feeding [5]. Therefore, the impact of secondary metabolites on aphids is limited. At the same time, in life-history strategies, trade-offs between reproduction and survival are pervasive. Salguero-Gómez et al. [49] found some species with high scores on both the fast–slow axis and the reproductive strategy axis. They attained greater lifetime reproductive success and frequency of reproduction due to a longer generation time. For example, Wu et al. [50] found that the reproduction of psyllids, *Bactericera gobica* Loginova, will increase on plants infested by mites *(Aceria pallida* Keifer). However, Li et al. [51] found that mites, *A. pallida,* can inhibit the growth and development of the psyllids.

Our previous study revealed that the development of *L. decempunctata* larvae was significantly accelerated, and their size and weight increased substantially when they were reared on plants infested with *A. gossypii* [27]. This intricate interspecific interaction may also be modulated by the SA and JA signaling pathways in plants. The SA signaling pathway activated by *A. gossypii* infestation suppresses the JA signaling pathway, which in turn promotes the development of *L. decempunctata*. In this study, we demonstrated that the aphid infestation activated the plant SA signaling pathway, concurrently promoteing their development and reproduction. Similarly, the application of the SA analogue BTH to host plants also exhibited a comparable promoting effect. Our results aligned with the findings of Xu et al. [52], who reported that exogenous SA treatment on *Nicotiana tabacum* L. enhanced the reproductive capacity of *B. tabaci*. Our research results confirmed that the phloem-sucking pest *A. gossypii* promoted its own growth and development by manipulating the plant SA signaling pathway, which might be the reason why aphids preferred to congregate for infestation and were more prone to outbreaks.

The activation of the SA and JA signaling pathways in plants often induces changes in the emission of volatile organic compounds (VOCs), thereby affecting the attraction of natural enemies [53,54]. For example, the infestation *A. thaliana* by *B. tabaci* activated the plant’s SA signaling pathway and then increased the release of the volatile substance β-myrcene, which subsequently attracted the parasitoid wasp *Encarsia formosa* Gahan to parasitize the pest [55]; infestation of *Brassica oleracea* L. by the chewing insect *Pieris rapae* L. triggered the plant’s JA signaling pathway, leading to an increase in both the diversity and quantity of volatile compounds released by the plant, which attracted more parasitic wasps, including *Cotesia glomerata* L., *Cotesia rubecula* Marshall, and *Diadegma semiclausum* (Hellén) to come prey on the pest [56]. Many studies have demonstrated that insects have evolved strategies to adapt to or manipulate plant defenses [3]. Both the watery and gel saliva of aphids contain proteins, enzymes, and metabolites that potentially interfere with plant defense pathways, resistance mechanisms, or both when phloem-sucking insects feed on plants, which in turn benefit the insect and facilitate sustained feeding from the sieve elements [57]. For example, effectors (Bt56) present in phloem-feeding insects have been shown to utilize the crosstalk between the SA and JA pathways to promote whitefly, *B. tabaci*, performance [52]. This study, along with our previous research [27], has elucidated the complex interspecific interactions between *A. gossypii* and *L. decempunctata*, providing critical insights into the disaster-causing and outbreak mechanisms of these two pests. Nevertheless, the impacts of their feeding behaviors on the VOCs of host plants and natural enemies, as well as the interference of insects with the defense pathways of plants, remain to be further explored.

## 5. Conclusions

Our results demonstrated that infestation by leaf beetles did not affect the development, reproduction, or survival of aphids. Conversely, the aphid infestation significantly promoted its own development and reproduction. The contents of JA and SA were increased dramatically in the plants infested by beetles and aphids, respectively. Furthermore, exogenous application of BTH promoted the development and reproduction of aphids; however, application of Me-JA suppressed the development of aphids. These findings revealed a complex and asymmetric interspecific relationship between these two pests, in which aphids indirectly facilitate their own development and reproduction through SA-mediated suppression of plant defenses.

## Data Availability

The raw data supporting the conclusions of this article will be made available by the authors on request.
